# Molecular Docking Studies and Anti-enzymatic Activities of Thai Mango Seed Kernel Extract Against Snake Venoms

**DOI:** 10.3390/molecules14041404

**Published:** 2009-03-31

**Authors:** Jiraporn Leanpolchareanchai, Pimolpan Pithayanukul, Rapepol Bavovada, Patchreenart Saparpakorn

**Affiliations:** 1 Department of Pharmacy, Faculty of Pharmacy, Mahidol University, Bangkok 10400, Thailand; E-mails: ry_110@yahoo.com (J.L.); pypph@mahidol.ac.th; pypph@hotmail.com (P.P.); 2 Department of Pharmaceutical Botany, Faculty of Pharmaceutical Sciences, Chulalongkorn University, Bangkok 10330, Thailand; E-mail: brapepol2@hotmail.com (R.B.); 3 Department of Chemistry, Faculty of Science, Kasetsart University, Bangkok 10900, Thailand; E-mail: patchareenart_s@yahoo.com (P.S.)

**Keywords:** *Calloselasma rhodostoma*, *Mangifera indica* L., Molecular docking study, *Naja naja kaouthia*, Pentagalloylglucopyranose, Polyphenols.

## Abstract

The ethanolic extract from seed kernels of Thai mango (MSKE) (*Mangifera indica* L. cv. ‘Fahlun’) (Anacardiaceae) and its major phenolic principle (pentagalloyl glucopyranose) exhibited dose-dependent inhibitory effects on enzymatic activities of phospholipase A_2_ (PLA_2_), hyaluronidase and L-amino acid oxidase (LAAO) of *Calloselasma rhodostoma* (CR) and *Naja naja kaouthia* (NK) venoms by *in vitro* tests. The anti-hemorrhagic and anti-dermonecrotic activities of MSKE against both venoms were clearly supported by *in vivo* tests. Molecular docking studies indicated that the phenolic molecules of the MSKE could selectively bind to the active sites or their proximity, or modify conserved residues that are critical for the catalysis of PLA_2_, and selectively bind to the LAAO binding pocket of both CR and NK venoms and thereby inhibit their enzymatic activities. The results imply a potential use of MSKE against snake venoms.

## 1. Introduction

Venomous snakebite is an important public health problem in remote areas of Thailand since the economic activities in these areas are mainly agricultural. *Naja naja kaouthia* Lesson (NK) (Thai cobra, Elapidae) causes the highest mortality due to snake envenomation in Thailand [1] and *Calloselasma rhodostoma* Kuhl (CR) (Malayan pit viper, Viperidae) causes the greatest incidence of envenomation in the country [2]. Among the many venomous snakes in Thailand, CR and NK venoms cause the most severe necrosis on the bite local tissue.

Snake venoms are complex mixtures comprised mainly of proteins and peptides possessing a variety of biological activities. Venom proteins have many diverse enzymatic activities [3]. Enzymes with hydrolytic activity such as phospholipase A_2_ (PLA_2_), hyaluronidase and L-amino acid oxidase (LAAO) are found in most snake venoms and are deeply involved in snake envenomation by inducing local effects such as severe inflammatory reactions, hemorrhage and necrosis of local tissues [4]. PLA_2_s (EC 3.1.1.4) are enzymes that specifically hydrolyzes the *sn*-2 ester bond of 3-*sn* membrane glycerophospholipids, generating lysophospholipids and free fatty acids, which themselves cause considerable erythrocyte membrane lytic and damage [5]. In addition, PLA_2_s exhibit wide varieties of pharmacological effects such as neurotoxicity, cardiotoxicity, myotoxicity, as well as necrotic, anticoagulant, hypotensive, hemolytic, hemorrhagic and edematogenic [6]. PLA_2_s from snake venoms are classified into groups I or II, based on their sequences and modes of disulphide pairings. Group I PLA_2_s are found in the venom of Elapidae snakes, whereas group II PLA_2_s are found in the venom of Viperidae snakes. PLA_2_s from different snake venoms share a high degree of homology in the amino acid sequence and enzymatic active sites. They hydrolyze phospholipids using a His-Asp doublet plus a conserved water molecule as a nucleophile, and a Ca^2+^ ion as a cofactor [7]. Hyaluronidase (EC 3.2.1.35), known as endoglycosidase, is frequently referred to a “spreading factor” owing to its ability to cause the distortion in the structural integrity of extracellular matrix in local tissues. This eventually results in the dissemination of target specific toxins. The process described herein is presumed to be the critical step in the enzyme-mediated spreading process [8]. LAAO (EC 1.4.3.2) is a flavoenzyme which catalyses the stereospecific oxidative deamination of an L-amino acid substrate to an α-keto acid along with the production of ammonia and hydrogen peroxide (H_2_O_2_) [9]. This enzyme induces apoptosis in mammalian endothelial cells possibly through the production of highly localized concentrations of H_2_O_2_[10]. In addition, Sakurai *et al*. [11] suggested that LAAO from NK venom also functions as a platelet aggregation inhibitor due to H_2_O_2_ formation. Recently, Pawelek *et al.* [12] demonstrated the high-resolution X-ray crystallographic structure of LAAO from CR venom, which indicated that snake venom LAAO is functionally a dimer. Each subunit consists of three domains: a FAD-binding domain, a substrate-binding domain and a helical domain. A funnel is formed by the interface between the substrate-binding and helical domains and provides substrate access to the active site.

Plant extracts constitute a rich source of bioactive compounds with a variety of pharmacological activities. Tannins from plants have been shown to interact with enzymes from snake venoms and act as an antidote [13,14,15,16]. The ethanolic extract of Thai mango seed kernels (MSKE) (*Mangifera indica* L. cv. ‘Fahlun’, Anacardiaceae) has a relatively high phenolic content and is composed of 1,2,3,4,6-penta-*O*-galloyl-β-D-glucopyranose (PGG) (61.28%), methyl gallate (MG) (0.68%) and gallic acid (GA) (0.44%) [17]. The extract and its phenolic principles have been shown to exhibit potent antioxidant, anti-tyrosinase, anti-inflammatory and hepatoprotective activities [17,18]. The aim of this study was to investigate the inhibitory effect of MSKE and its isolated phenolic principles on the enzymatic activities of PLA_2_, hyaluronidase and LAAO of CR and NK venoms by *in vitro* tests. The inhibitory effects of MSKE on the activities of these enzymes were also observed by following *in vivo* tests on the anti-hemorrhagic and anti-dermonecrotic activities of CR and NK venoms, respectively. Molecular docking studies using the Gold v3.2 program were carried out to investigate the binding mode of the extract’s principles to the enzymes. 

## 2. Results and Discussion

### 2.1. In vitro tests for the inhibition of enzymatic activities

#### 2.1.1. PLA2

**Figure 1:**
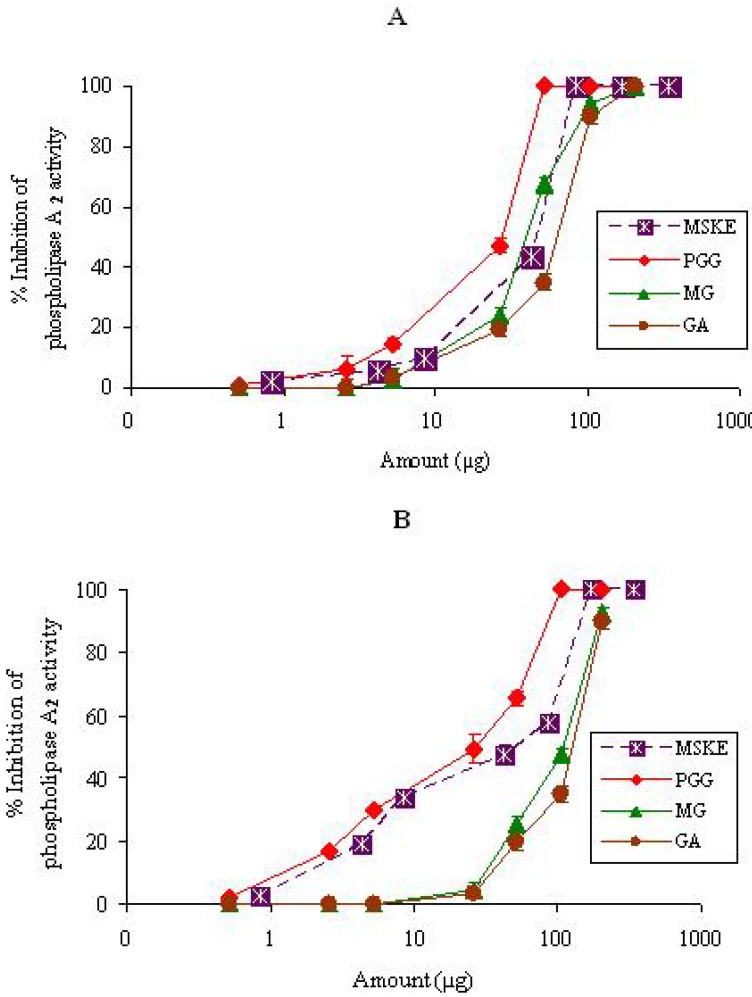
Semi-log plot of inhibition of PLA_2_ activity of CR (17.1 µg) (A) and NK (3.4 µg) (B) venoms by MSKE and its phenolic principles (GA, MG and PGG). Values are mean ± S.E.M. of duplicate experiments.

CR (17.1 µg) and NK (3.4 µg) venoms produced clear zone diameters of 21.1 ± 0.3 mm and 20.6 ± 0.3 mm, respectively, on agarose-erythrocyte egg yolk gel plate and these clear zones were selected as the minimum indirect hemolytic dose for each venom. From the indirect PLA_2_ assay, the results revealed that the enzyme from both venoms exhibited indirect hemolytic activity. This was probably due to the activity of PLA_2_s in the hydrolysis of lecithins to lysolecithins and lysis of the cell membranes of red blood cells [19]. Figure 1 shows the log dose-response curves of MSKE and its phenolic principles (GA, MG and PGG) on the inhibition of PLA_2_s of CR and NK venoms. It can be seen that MSKE and its phenolic principles clearly showed a dose-dependent inhibitory activity against PLA_2_s of both venoms. The order of potency as judged by the half-effective dose (ED_50_) was PGG (25.6 ± 1.3 µg) > MG (42.9 ± 2.3 µg) > MSKE (44.4 ± 0.4 µg) > GA (66.0 ± 2.5 µg) for CR venom, and PGG (30.2 ± 2.4 µg) > MSKE (57.9 ± 0.5 µg) > MG (108.7 ± 2.0 µg) > GA (123.5 ± 2.5 µg) for NK venom (Table 1). The extract, GA, MG and PGG alone and solvent systems had no effect on this study. Of these results implied that the inhibition of PLA_2_s of both venoms by MSKE may result in the prevention of red blood cell rupture.

**Table 1:** The constituents and contents of Thai MSKE; the effective dose giving 50% inhibition (ED_50_) of venom enzymatic activities of MSKE and its three isolated pure compounds. The values represent means ± S.E.M. (n = 2).

MSKE and its isolated principles	Contents	PLA_2_ (ED_50_, µg)		Hyaluronidase (ED_50_, µg)		LAAO (ED_50_, µg)	
	(mg/g dry weight)	CR venom	NK venom	CR venom	NK venom	CR venom	NK venom
MSKE	-	44.4 ± 0.4	57.9 ± 0.5	6.0 ± 1.9	5.0 ± 2.6	1.5 ± 3.2	4.0 ± 1.7
GA	4.40 ± 0.05	66.0 ± 2.5	123.5 ± 2.5	16.0 ± 2.5	14.8 ± 1.4	1.7 ± 3.4	4.5 ± 2.8
MG	6.80 ± 0.02	42.9 ± 2.3	108.7 ± 2.0	5.9 ± 2.4	8.6 ± 2.1	2.3 ± 2.4	4.4 ± 2.0
PGG	612.80 ± 34.80	25.6 ± 1.3	30.2 ± 2.4	3.4 ± 2.4	2.6 ± 1.7	0.9 ± 2.4	2.2 ± 2.0

#### 2.1.2. Hyaluronidase

**Figure 2:**
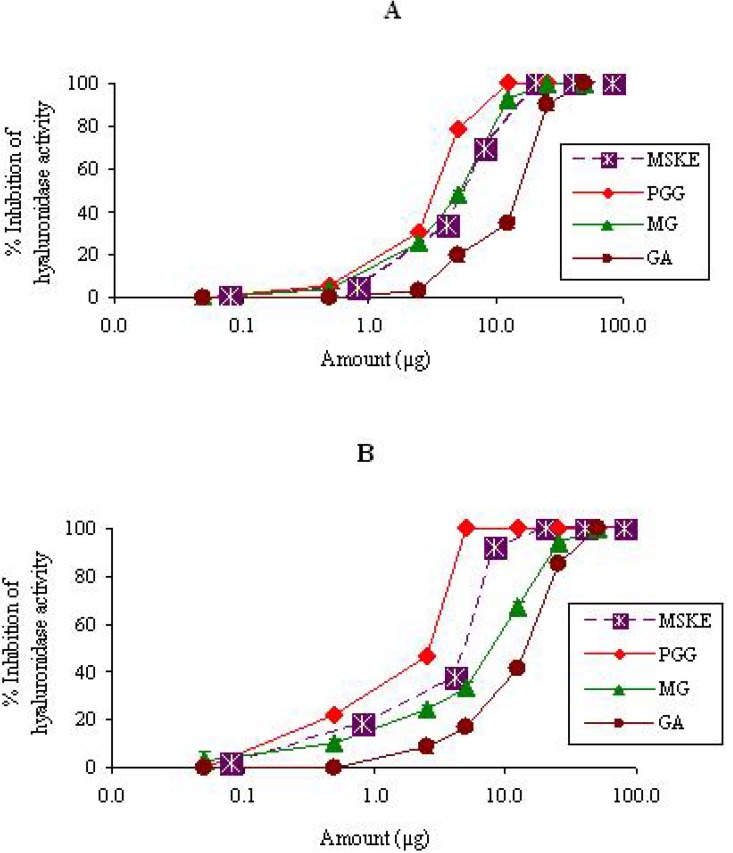
Semi-log plot of inhibition of hyaluronidase activity of CR (250 µg) (A) and NK (250 µg) (B) venoms by MSKE and its phenolic principles (GA, MG and PGG). Values are mean ± S.E.M. of duplicate experiments.

MSKE and its phenolic principles (GA, MG and PGG) inhibited the activity of hyaluronidase in CR and NK venoms in a dose-dependent manner (Figure 2). The order of potency as judged by the half-effective dose (ED_50_) was PGG (3.4 ± 2.4 µg) > MG (5.9 ± 2.4 µg) > MSKE (6.0 ± 1.9 µg) > GA (16.0 ± 2.5 µg) for CR venom, and PGG (2.6 ± 1.7 µg) > MSKE (5.0 ± 2.6 µg) > MG (8.6 ± 2.1 µg) > GA (14.8 ± 1.4 µg) for NK venom (Table 1). The inhibition of this enzymatic activity by MSKE may contribute to the prevention of extracellular matrix degradation, and thereby decrease the diffusion of toxins through tissues.

#### 2.1.3. LAAO

**Figure 3:**
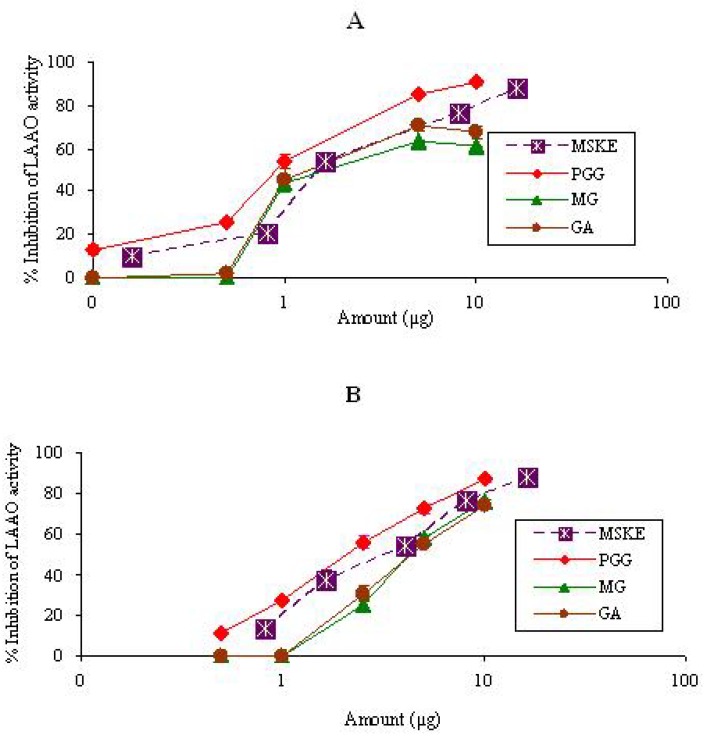
Semi-log plot of inhibition of LAAO activity of CR (50 µg) (A) and NK (50 µg) (B) venoms by MSKE and its phenolic principles (GA, MG and PGG). Values are mean ± S.E.M. of duplicate experiments.

MSKE and its phenolic principles (GA, MG and PGG) inhibited the LAAO activity of both venoms in a dose-dependent manner (Figure 3). The order of potency as judged by the half-effective dose (ED_50_) was PGG (0.9 ± 2.4 µg) > MSKE (1.5 ± 3.2 µg) > GA (1.7 ± 3.4 µg) > MG (2.3 ± 2.4 µg) for CR venom, and PGG (2.2 ± 2.0 µg) > MSKE (4.0 ± 1.7 µg) > MG (4.4 ± 2.0 µg) > GA (4.5 ± 2.8 µg) for NK venom (Table 1). In this assay, H_2_O_2_ generated by the enzyme of both venoms was used by horseradish peroxidase to oxidize *o*-dianisidine to the radical cation, which was monitored at 436 nm using a spectrophotometer. Therefore, the inhibition of LAAO activity of CR and NK venoms by the MSKE might presumably play a role in the prevention of highly localized concentrations of generated H_2_O_2_ in the victim’s tissues, since this snake venom enzyme can induce necrosis and apoptosis in mammalian endothelial cells and consequently cause hemorrhage, possibly through the production of highly localized concentrations of H_2_O_2_[10]. The inhibition of LAAO activity by the MSKE may contribute to the prevention of hemorrhage and necrosis.

It is seen from Figure 1, Figure 2 and Figure 3 and Table 1 that MSKE and its phenolic principles (PGG, MG and GA) exhibited dose-dependent inhibitory effects on PLA_2_, hyaluronidase and LAAO activities of both venoms. These results indicated that the anti-enzymatic potency of MSKE may be attributed to its major principle (PGG) and other unidentified constituents, since PGG exerted its effect at the lowest ED_50_ value and had the highest percentage content (61.28%) within the MSKE compared with GA (0.44%) and MG (0.68%). It could be noted that although GA and MG inhibited these enzymatic activities of both venoms, their percentage content within the MSKE were very low. Therefore, GA and MG may only possess a negligible effect compared with PGG. It has been reported that other plant extracts also contain compounds that capable of neutralizing PLA_2_, hyaluronidase and LAAO activities of snake venoms [20,21,22]. The neutralization of these enzymes not only prevents the early effects of envenomation (local tissue damage, paralysis and hypotension), but also limits the systemic effects by reducing the distribution of toxin.

### 2.2. In vivo tests

#### 2.2.1. Inhibition of hemorrhagic and dermonecrotic activities

**Figure 4:**
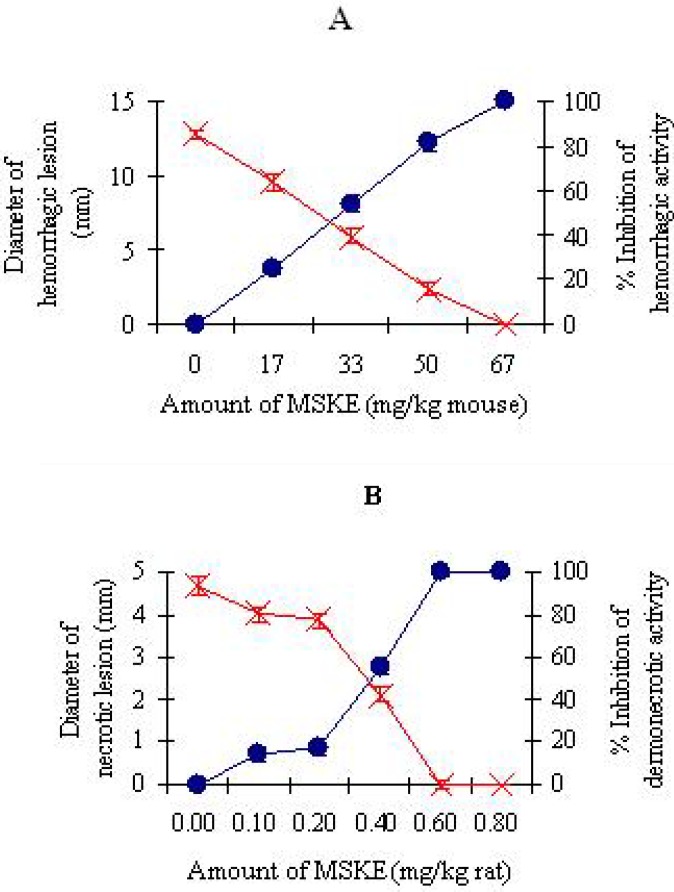
Inhibition of hemorrhagic activity of CR venom (61.2 µg/mouse) in mice (n = 8) (A) and inhibition of dermonecrotic activity of NK venom (63 µg/rat) in rats (n = 4) (B) by MSKE. Diameters of hemorrhagic and necrotic lesions (×). Percentage inhibition of hemorrhagic and necrotic activities (●).

The minimum hemorrhagic dose (MHD) of CR venom was determined to be 20.4 µg/mouse, while the venom at three MHDs-induced hemorrhagic lesions with a diameter of 13.4 ± 0.4 mm. The MSKE significantly (*P* < 0.05) diminished the diameters of hemorrhagic lesions induced by three MHDs of CR venom in a dose-dependent manner, and the hemorrhagic activity could be completely inhibited at the extract amount of 67 mg/kg mouse (Figure 4A). The extract and solvent systems used did not induce hemorrhagic lesion in mice. The minimum necrotizing dose (MND) of NK venom was determined to be 63 µg/rat, which induced a necrotic lesion with a diameter of 4.7 ± 0.1 mm. Figure 4B shows the percentage inhibition of dermonecrotic activity of NK venom by MSKE which was dose-dependent. The extract with an amount of 0.60 mg/kg rat was effective in completely protecting rats from the dermonecrotic activity of one MND of NK venom (*P* < 0.05). Solvent and the extract alone did not induce necrotic lesion in rats.

### 2.3. Molecular modeling

#### 2.3.1. Docking into PLA_2_-CR and PLA_2_-NK structures

**Figure 5:**
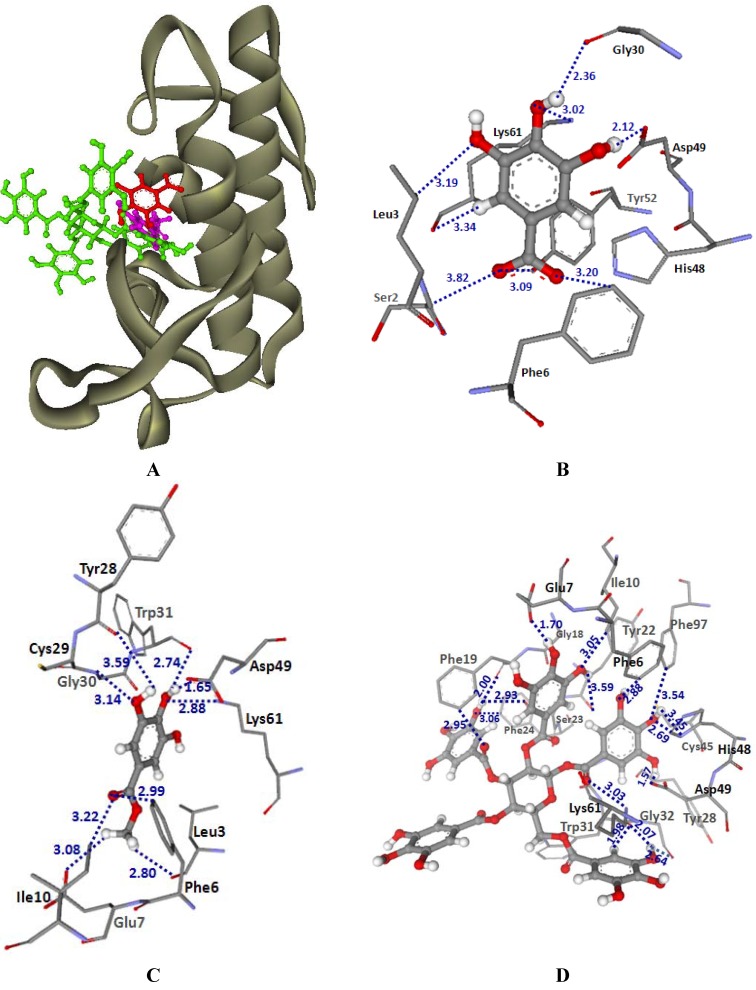
(A) Docked conformation of ligand structures in PLA_2_-CR structure (GA: Red, MG: Margenta and PGG: Green). (B–D) Distances (in Å) between residues in PLA_2_-CR binding pocket and ligands: GA (B), MG (C) and PGG (D).

**Figure 6:**
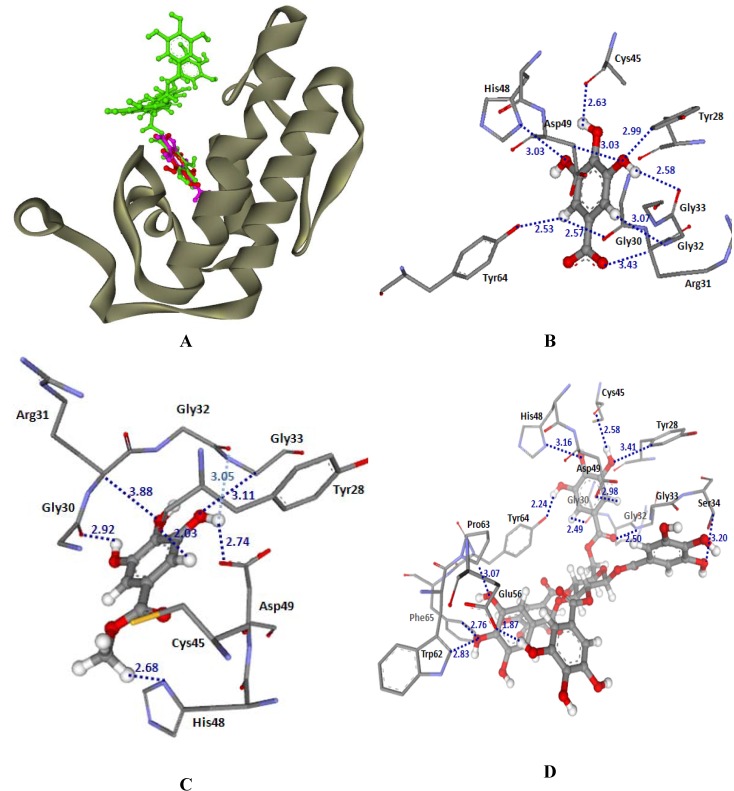
(A) Docked conformation of ligand structures in PLA_2_-NK structure (GA: Red, MG: Margenta and PGG: Green). (B-D) Distances (in Å) between residues in PLA_2_-NK binding pocket and ligands: GA (B), MG (C) and PGG (D).

The results of molecular modeling using a molecular docking method revealed the possible molecular orientation of MSKE constituents (GA, MG and PGG) in the PLA_2_ binding pocket of CR and NK venoms (Figure 5 and and Table 2). GoldScores revealed that PGG was bound tighter than GA and MG in their binding pockets of CR and NK venoms. The binding orientations of GA, MG and PGG in the PLA_2_-CR structure are shown in Figure 5A. Strong H-bonding interactions from GA, MG and PGG were found between a hydrogen atom of the hydroxyl group of ligands and an oxygen atom of the carboxylate group of Asp49. Moreover, common H-bonding interactions with Phe6, Gly30 and Lys61 were shown in the docked GA, MG and PGG. GA had a GoldScore of 37.55 and other H-bonding interactions were also formed with Ser2, Leu3 and Tyr52 ( Figure 5B). In Figure 5C, docked MG, with a GoldScore of 37.49, revealed other H-bonding interactions with Leu3, Glu7, Ile10, Tyr28, Cys29 and Trp31. Docked PGG had a GoldScore of 77.26 and revealed H-bonding interactions with Phe6, Glu7, Ile10, Gly18, Phe19, Tyr22, Ser23, Phe24, Tyr28, Gly30, Trp31, Gly32, Cys45, His48, Asp49, Lys61 and Phe97 (Figure 5D). The docked conformations of GA, MG and PGG in PLA_2_-NK structure are shown in Figure 6. Their GoldScores were 35.52, 34.75 and 64.92, respectively. The docked conformation of GA, MG and PGG showed common H-bonding interactions with Tyr28, Gly30, Arg31, Gly32, Gly33, Cys45, His48, Asp49 and Tyr64. Additional H-bonding with Ser34, Glu56, Trp62, Pro63 and Phe65 were also formed with the docked conformation of PGG. It can be seen from Figure 5 and Figure 6 that the tannin principles of MSKE could form hydrogen bonds with active site residue His48 N^δ1^ and Asp49 O^δ1^ of the PLA_2_ from CR and NK venoms. His48 and Asp49 amino acids are essential for enzymatic activity in the calcium-dependent PLA_2_s; if the inhibitors are bonded to these amino acids the PLA_2_ catalytic efficiency can be changed [7,23]. Furthermore, the tannins of MSKE also form important hydrogen bonds with the carbonyl oxygen atoms of the residues involved in calcium coordination (Tyr28, Gly30, Gly32 and Asp49) [23]. Therefore, the results of molecular docking indicated that the phenolic molecules of MSKE could selectively bind to the active sites or their proximity, or to modify conserved residues that are critical to the catalysis of PLA_2_ enzymes. In addition, a previous study found that MSKE was a natural chelator [17] and it is well known that PLA_2_s depend on Ca^2^^+^ to exert its action. This confirmed the presence of a selective mechanism for MSKE against PLA_2_ activity in CR and NK venoms. 

**Table 2:** GoldScores of ligands docked into LAAO-CR, PLA_2_-CR and PLA_2_-NK structures.

	GoldScore		
MSKE constituents	LAAO	PLA_2_	
	CR venom	CR venom	NK venom
GA	38.95	37.55	35.52
MG	39.71	37.49	34.75
PGG	69.58	77.26	64.92

#### 2.3.2. Docking into LAAO-CR structure

**Figure 7:**
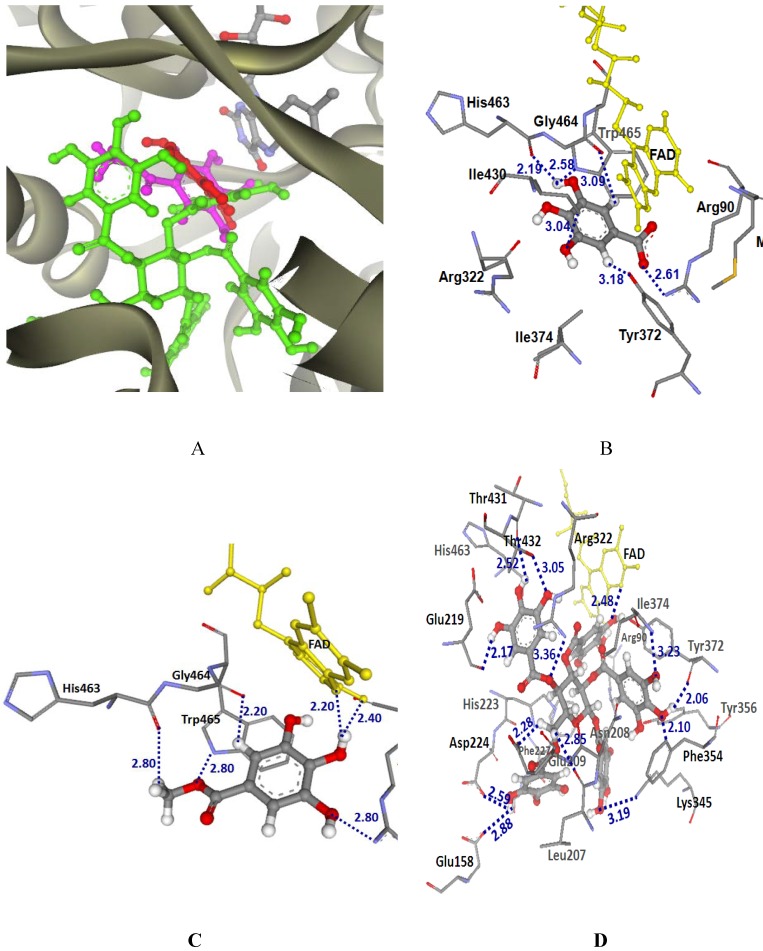
(A) Docked conformation of ligand structures in LAAO-CR structure (GA: Red, MG: Margenta and PGG: Green). (B–D) Distances (in Å) between residues in LAAO-CR binding pocket and ligands: GA (B), MG (C) and PGG (D).

The possible molecular orientation of the MSKE constituents (GA, MG and PGG) in the LAAO binding pocket of CR venom is shown in Figure 7A. Their GoldScores (Table 2) were 38.95, 39.71 and 69.58, respectively. It was proposed that PGG was bound in the LAAO binding pocket of CR venom tighter than GA and MG. From their docked conformation in LAAO-CR structure, common H-bonding interaction of GA, MG and PGG were found with Arg90 and His463. GA also formed H-bonding interaction with Tyr372, Ile430, Gly464 and Trp465, as shown in Figure 7B. Moreover, a H-π interaction was found between GA and phenyl ring of Trp465 with a distance about 3 Å. In the case of docked MG, it revealed H-bonding interactions with Gly464, Trp465, and FAD and a π-π interaction with Trp465 (Figure 7C). The docked PGG showed H-bonding interactions with Leu207, Asn208, Glu209, Glu219, His223, Asp224, Phe227, Ala228, Arg322, Lys345, Phe354, Tyr356, Tyr372, Ile374, Thr431, Thr432 and FAD (Figure 7D). The interactions found between the amino acid residues of LAAO and tannins in this study agreed well with the crystal structures of LAAO complexed with citrate and with *o*-aminobenzoate [12]. Pawelek *et al.* [12] reported the amino acid residues of LAAO were involved in the binding of citrate and *o*-aminobenzoate; *i.e.* Leu51, Arg90, Asn208, His223, Asp224, Arg283, Gly313, Tyr372, Ile430 and Gly464. It can be seen from Figure 7 that the tannins of MSKE could form hydrogen bonds with amino acid residues in the substrate-binding domain (Arg90, His223, Asp224, Phe227, Ala228, Lys345, Phe354, Tyr356, Tyr372 and Ile374) of the LAAO from CR venom. These results indicated the presence of a selective mechanism of extract against LAAO activity in CR venom, since a protein database search revealed that the N-terminal sequence of LAAO from NK venom had the highest similarity score to LAAO from CR venom and had similarities to several other snake venom LAAOs [11]. Similar to CR venom, the phenolic compounds of the MSKE could therefore possibly bind to the LAAO binding pocket of NK venom and thereby inhibited the LAAO activity.

The *in vivo* inhibition of viper venom-induced hemorrhagic and cobra venom-induced dermonecrotic actions by MSKE is proposed. The inhibition was due firstly to a non-selective mechanism as a result of complexation between the venom proteins and tannins in the extract at the injection site [24] and to a selective mechanism where the active sites of the enzymes (PLA_2_ and LAAO) of CR and NK venoms were blocked by the phenolic principles of MSKE as demonstrated in the molecular modeling study (Figure 5, Figure 6, and Figure 7). In addition, the MSKE has been reported to demonstrate chelating property with ferrous metal ions [17]; therefore, the authors will investigate further on the potential of MSKE in chelating the venom’s hemorrhagic Zn^2+^-metalloproteinases since these enzymes presumably act by destroying the collagenous basement membrane and other connective tissue collagen with consequent weakening of blood vessel stability causing the hemorrhagic effect [25]. The molecular docking method of the phenolic principles of the MSKE with the crystal structure of venom metalloproteinases will also be evaluated further.

## 3. Conclusions

This study shows a beneficial relationship between the *in vitro* anti-enzymatic activities and *in vivo* anti-hemorrhagic and anti-dermonecrotic activities of MSKE and its isolated principles against CR and NK venoms. The results were clearly supported by molecular docking studies which indicated that the phenolic principles of MSKE could selectively block the active sites of PLA_2_ and LAAO of both venoms and PGG were bound tighter than GA and MG. These results imply that the anti-enzymatic potency of MSKE may be attributed by its major principle (PGG) since PGG exerted its effect at the lowest value of ED_50_ with the highest GoldScores and had the highest percentage content within the MSKE compared with GA and MG.

## 4. Experimental

### 4.1. Animals and venoms

Male Swiss albino mice (25–30 g) and male Sprague-Dawley rats (250–300 g) were used for the assays of anti-hemorrhagic and anti-dermonecrotic activities, respectively. Experiments were carried out according to the guidelines set by Mahidol University Animal Care and Use Committee (PY-ACUC). Lyophilized CR and NK venoms were obtained from the Queen Saovabha Memorial Institute, Thai Red Cross Society, Bangkok, Thailand. 

### 4.2. Chemicals

Gallic acid (GA; ≥ 98%) and methyl gallate (MG; ≥ 98%) were purchased from Fluka (Buchs, Switzerland). Pentagalloylglucopyranose (PGG; ≥ 95%) was obtained from Endotherm GmbH (Germany). *o*-Dianisidine (≥ 97%), horseradish peroxidase (987 U/mg solid), hyaluronic acid (≥ 95%) and L-leucine (≥ 98%) were purchased from Sigma Chemical (St. Louis, MO, USA). All reagents used in the *in vitro* and *in vivo* experiments were obtained from commercial sources and were of analytical grade.

### 4.3. Plant material and extraction

Fully grown unripened Thai mango fruits (*Mangifera indica* L. cv. ‘Fahlun’) were purchased from a local market. A voucher specimen (R.B. 20007) was deposited at the Museum of Natural Medicine, Faculty of Pharmaceutical Sciences, Chulalongkorn University, Bangkok, Thailand. The ethanolic extract from fresh mango seed kernels was obtained by chopping and homogenizing the seed kernels in a blender, using hot ethanol (80 °C) as an extracting solvent at a ratio of seed kernels/solvent = 1:2 (w/v) for 10 min at room temperature (30 °C) prior to centrifugation at 2000 rpm for 15 min in a Hattich Roto magna^®^. The extraction was performed three times. The ethanolic extracts were filtered, combined and concentrated in a rotary evaporator (Büchi Rotavapor R-200) at 40 °C. The extract was defatted with hexane, evaporated under reduced pressure, and then freeze-dried to afford a crude mango seed kernel extract (MSKE) with a yield of 9.36% (w/w). 

### 4.4. Standardization

GA, MG and PGG were used as chemical markers and the calibration curves for each compound were obtained by densitometric scanning of different quantities of the chemical marker bands on developed chromatographic plates. An aliquot of the crude extract (MSKE) (8 μL, 25 mg/mL) was applied along with serial amounts of the chemical marker stock solution. The thin-layer chromatographic (TLC) plates were developed in a pre-saturated twin trough glass tank using CHCl_3_/MeOH/EtOAc/ethyl methyl ketone (6:1.6:2:2) with five drops of formic acid as the mobile phase for GA and MG and CHCl_3_/EtOH/formic acid (3:5:1) for PGG. The developed TLC plates were scanned at 286 nm and the amount of each compound (GA 4.4, MG 6.8 and PGG 612.8 mg/g dry weight) in MSKE was calculated from the calibration curves [17].

### 4.5. In vitro tests

#### 4.5.1. Inhibition of PLA_2_ activity

PLA_2_ activity in CR and NK venoms was measured using an indirect hemolytic assay on agarose-erythrocyte-egg yolk gel plates to define the minimum indirect hemolytic dose (MIHD) [26], the dose of venom that induced a hemolysis halo of 20 mm diameter after incubation for 20 h at 37 °C. The inhibition of PLA_2_ activity by MSKE and its isolated phenolic principles was determined against one MIHD of CR and NK venoms. Test solutions and the venoms (0.05 mL each) were pre-incubated for 1 h at 37 °C. After centrifugation at 10,000 rpm for 10 min, the supernatant (20 µL) was tested for PLA_2_ activity. The anti-PLA_2_ potential of the extract and its phenolic principles was presented as percent inhibition of the enzymatic activity versus dose, in which 100% inhibition should produce no clear zone. ED_50_, the effective dose that PLA_2_ activity of the venoms was reduced by 50%, was calculated. 

#### 4.5.2. Inhibition of hyaluronidase activity

Hyaluronidase activity was determined according to the methods of Ferrante [27] and Yingprasertchai *et al.* [28] with slight modification. CR or NK venom (25-1,000 µg) in PBS (pH 7.4) was incubated with hyaluronic acid (50 μg) in 0.2 M sodium acetate buffer (1 mL, pH 5) that contained 0.15 M NaCl for 25 min at 37 °C. The reaction was stopped by adding 2.5% cetyltrimethylammonium bromide in 2% NaOH (2 mL). The optical density at 400 nm of each sample was read after 30 min with a Shimadzu UV-VIS spectrophotometer UV-160A against blank. Turbidity-reducing activity was expressed as the percentage of the remaining hyaluronic acid, taking the absorbance of a tube to which no enzyme was added as 100%. The venom concentration that produced a reduction in turbidity of ~50% was used in inhibition experiments. The inhibition of venom hyaluronidase activity by MSKE and its isolated phenolic principles was determined after pre-incubating an equal volume (100 µL) of test sample (with different amounts) in acetate buffer with CR or NK venom in PBS (2,500 µg/mL) for 30 min at 37 °C, after which hyaluronidase activity was measured. Blanks containing only the buffer and test sample were run in parallel. The anti-hyaluronidase potential of the extract and its phenolic principles was presented as percent inhibition of the enzymatic activity versus dose. ED_50,_ the effective dose that hyaluronidase activity of the venoms was reduced by 50%, was calculated.

#### 4.5.3. Inhibition of LAAO activity

LAAO activity of CR and NK venoms was determined by using an enzyme-coupled assay [29]. The assay mixture contained 0.025 mL 0.014% peroxidase and 0.2 M triethanolamine buffer (1 mL, pH 7.6) that contained 0.1% L-leucine and 0.0065% *o*-dianisidine. After incubation at 25 °C for 3 min, 0.1 mL CR or NK venom in PBS (1 mg/mL) was added, and an increase in absorbance at 436 nm (Shimadzu UV-VIS spectrophotometer UV-160A) was measured against blank. Enzymatic activity was calculated from the linear portion of the time–absorbance plot, where one unit of enzymatic activity was defined as the amount of enzyme that caused an increase of 0.001 absorbance units per minute. The percent inhibition of LAAO activity by MSKE and its isolated phenolic principles was evaluated by pre-incubating CR or NK venom (0.1 mL, 1 mg/mL) with test sample in sterile water for injection (SWI) (0.1 mL) for 1 h at 37 °C. After pre-incubation, the mixture was centrifuged at 10,000 rpm for 5 min, and supernatant (0.1 mL) was evaluated for LAAO activity. Venoms, test sample, and solvents alone were used as controls. The anti-LAAO potential of the extract and its phenolic principles was presented as percent inhibition of the enzymatic activity versus dose. ED_50,_ the effective dose that LAAO activity of the venoms was reduced by 50%, was calculated.

### 4.6. In vivo tests

#### 4.6.1. Inhibition of hemorrhagic activity

Hemorrhagic activity of CR venom was determined according to the method of Kondo *et al.* [30]. The minimum hemorrhagic dose (MHD) of CR venom was established by injecting 40 µL of different amounts of venom in sterile PBS (pH 7.2) intradermally (i.d.), into two separately marked positions on the shaved dorsal skin of unanesthesized mice (n = 8). After 90 minutes, mice were killed with an overdose of ether, the dorsal skin was removed and the diameter of the hemorrhagic lesion on the inner surface of the skin was measured using calipers and background illumination. The mean diameter of the hemorrhagic lesion was calculated for each venom dose. A dose-response curve for venom dose and hemorrhagic lesion diameter was plotted, and the MHD was recorded as the dose that caused a lesion diameter of 10 mm. The anti-hemorrhagic potential of MSKE was determined against three MHDs of CR venom in mice (n = 8) according to the modified methods of Yingprasertchai *et al.* [28] and Gutiérrez *et al.* [31]. Twenty microliters of venom in sterile PBS (3.06 μg/μL) was injected (i.d.) into the shaved dorsal skin of mice, immediately followed by test sample in SWI (20 µL) at the same injection site. After 90 minutes, mice were killed by ether overdose. Dorsal skin was removed and the diameter of the hemorrhagic lesion on the inner surface of the skin was measured. Sterile PBS, SWI, venom and MSKE alone were used as controls.

#### 4.6.2. Inhibition of dermonecrotic activity

The methods of Kondo *et al.* [30] and Theakston and Reid [32] were used to determine the minimum necrotizing dose (MND) of NK venom in rats (n = 4) which is defined as the smallest amount of venom that could cause a necrotic lesion of 5 mm in diameter on the inner dorsal skin of the rat 72 h after intradermal injection. The anti-dermonecrotic potential of MSKE was determined against one MND of NK venom. The venom in sterile PBS (40 µL, 1.57 μg/μL) was injected i.d. into the shaved dorsal skin of rats (n = 4), immediately followed by test sample (40 µL) at the same injection site. Seventy-two hours after injection, rats were killed by ether overdose. The dorsal skin was removed, and the necrotic lesion on the inner surface of the skin was measured. Sterile PBS, venom, and MSKE alone were used as controls. 

### 4.7. Molecular modeling

**Figure 8:**
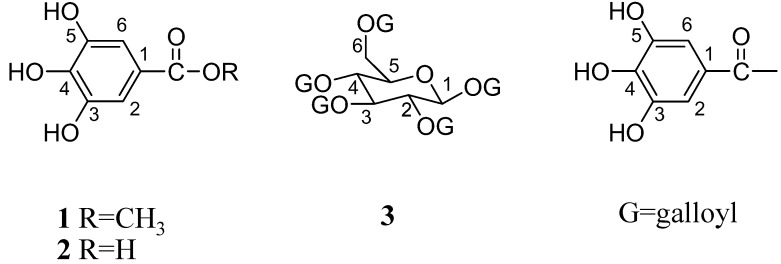
Chemical structures of three major constituents of Thai mango seed kernel extract: MG (**1**), GA (**2**) and PGG (**3**).

Structures of gallic acid (GA), methyl gallate (MG) and 1,2,3,4,6-penta-*O*-galloyl-β-d-glucopyranose (PGG) (Figure 8) were constructed and optimized at the HF/3-21G level of theory using Gaussian 03 program [33]. For LAAO structure, the X-ray structure of LAAO from *Calloselasma rhodostoma* complexed with citrate was obtained from the Protein Data Bank (PDB code 1f8r) [12]. Protein structure and Flavin Adenine Dinucleotide (FAD) were selected and added hydrogen atoms by using SYBYL version 7.2 program (TRIPOS, Assoc., Inc.: St. Louis, MO). The three-dimensional structures of PLA_2 _ from CR and NK venoms were built by homology modeling technique using Geno3D web server [34]. Geno3D web server used distance geometry, simulated annealing and energy minimization algorithms to build the protein 3D model. After that, the structures were checked the quality of geometry by using PROCHECK [35]. For homology modeling of PLA_2_ structures from CR venom, the sequence of CRV-S1E6a [36] was based on the structure of PLA_­2_­ from venom of *Agkistridon piscivorus piscivorus* (PDB code 1vap) which has the 65% sequence identity. Homology modeling of amino acid sequences of PLA_­2_­ (CM-III) from NK venom [37] was based on the structure of PLA_­2_­ from *Naja naja naja* venom (PDB code 1a3d), which had a high sequence identity of 90%. Hydrogen atoms were added to all structures by using the SYBYL 7.2 program.

The molecular docking method was done using Gold version 3.2 program [38] to investigate the binding orientation of GA, MG and PGG in LAAO from CR venom, and PLA_2_ from CR and NK venoms. The radius of the binding site was set to 10 Å. For LAAO structure, the binding site was defined by using the center of mass of citrate which is in the active site. For both PLA_2_ structures, the possible binding sites were predicted by using PASS (Putative Active Sites with Spheres) program [39]. PASS identified the positions likely to represent binding sites based upon the size, shape and burial extent of these volumes. The most possible position representing the binding site from PASS, as called calcium-binding loop in the PLA_2_ structure [40], was used to define the binding site in Gold program. For setting the genetic algorithm parameters, the default parameters of automatic settings were used. The GoldScore fitness function was used to determine the fitness score. The GoldScore fitness function was used to determine the fitness score, as shown in GoldScore.

### 4.8. Statistical analysis

Results were expressed as mean ± S.E.M. for two determinations. Statistical analysis was carried out using SPSS 13.0 for Windows. Significant differences (*P* < 0.05) between means were assessed by one-way ANOVA, followed by Tukey’s honesty significant difference test or Dunnett’s T3 test for multiple comparisons.
